# Comparison of artificial graft versus autograft in anterior cruciate ligament reconstruction: a meta-analysis

**DOI:** 10.1186/s12891-017-1672-4

**Published:** 2017-07-19

**Authors:** Zhen-Yu Jia, Chen Zhang, Shi-qi Cao, Chen-chen Xue, Tian-ze Liu, Xuan Huang, Wei-Dong Xu

**Affiliations:** 1 Department of Orthopedics, Changhai Hospital, Second Military Medical University, Shanghai, China; 2 Department of Joint Surgery and Sports Medicine, Changzheng Hospital, Shanghai, China

**Keywords:** Artificial ligament, Autograft, Anterior cruciate ligament, Reconstruction

## Abstract

**Background:**

Critically evaluation and summarization for the outcomes between autografts and artificial grafts using in anterior cruciate ligament (ACL) reconstruction have not been performed currently. The purpose of this study is to compare the clinical outcomes between artificial ligaments and autografts at a short- to mid-term follow-up.

**Methods:**

A computerized search of the databases was conducted including Medline, Embase, and the Cochrane library. Only prospective or retrospective comparative studies with a minimum 2-year follow-up and a minimum sample size of 15 for each group were considered for inclusion. Two independent reviewers performed data extraction and methodological quality assessment. A Mantel-Haenszel analysis was used for pooling of results. Sensitivity analysis was performed in order to maintain the stability of results.

**Results:**

Seven studies were included in this study. The total sample size was 403 (autograft group: 206 patients; synthetic graft group: 197 patients). Four studies were randomized controlled trials. Two studies were retrospective comparative studies and one study was non-randomized prospective comparative study. In terms of instrumented laxity, patient-oriented outcomes and complications, no significant difference was occurred between new artificial ligaments and autografts. But the results of IKDC grades and instrumented laxity were worsen in early artificial ligaments compared to autografts.

**Conclusions:**

The outcomes of new generation of artificial ligaments are similar to autografts at a short- to mid-term follow-up. However, the early artificial ligaments are not suggested for ACL reconstruction compared to autografts.

## Background

Anterior cruciate ligament (ACL) injury is a main cause of recurrent knee instability and may result in secondary damages to other structures of the knee, such as meniscal tears and articular cartilage degeneration [1]. Currently, ACL reconstruction is the gold-standard surgical technique for ACL injury [2]. Reconstruction can be performed by using autograft, allograft or synthetic graft [3]. Despite the vast amount of researches, there still have a great deal of debates concentrating on the clinical outcomes of using different grafts in ACL reconstruction.

Autograft is a well-recognized and widely used material for ACL reconstruction due to a good graft stability and a well return to high-level sports [4]. And bone-patella tendon-bone (BPTB) autograft has historically served as the gold standard for ACL reconstruction based not only on widespread global use but also as the first autograft option. Reconstruction with synthetic grafts has the advantage of eliminating both the donor-site morbidity and disease transmission with fast rehabilitation [5]. High graft failures, no so-called ligamentization and severe synovitis have been reported as major disadvantages of synthetic grafts [6–8].

A few conventional narrative reviews have addressed related issues about the graft selection for ACL reconstruction [9–12]. Firm conclusions regarding the clinical outcomes with autografts or synthetic grafts cannot be drawn from those narrative reviews due to some inherent bias. Moreover, there have already been systematic reviews and meta-analysis which compared the clinical outcomes between allografts and autografts using in ACL reconstruction [13–16]. Critically evaluation and summarization for the outcomes between autografts and synthetic grafts using in ACL reconstruction have not been performed currently.

Using the best available evidence, the purpose of this research is to compare synthetic grafts with autografts in ACL reconstruction by evaluation the clinical outcomes including the results of instrumented laxity, patient-oriented outcomes, complications and graft failures.

## Methods

### Searching strategy

This research was conducted following the Preferred Reporting Items for Systematic Reviews and Meta-Analyses (PRISMA) statement [17]. Two researchers searched the international databases independently up to December 30^th^, 2016, including Medline, Embase, and the Cochrane library. OpenGrey, the World Health Organization International Clinical Trials Registry Platform, the International Standard Randomised Controlled Trial Number (ISRCTN) registry, and Current Controlled Trials were searched to review the trial registry and grey literature. There was no restriction to years of publication and languages.

### Eligibility criteria

Eligibility criteria were as follows: 1) a clinical study with a prospective or retrospective comparative design (Level of Evidence I, II, or III) [18]; 2) patients with no limitation of race and sex undergoing primary ACL reconstruction; 3) a study of ACL reconstruction comparing autografts with synthetic grafts and no restriction for types; 4) the outcomes being evaluated including physical examinations, complications, or patient-oriented outcomes etc.; 5) at least 2 years follow-ups; 6) at least 15 sample size for each group [15]. Knee laxity assessments included the arthrometer test and physical examinations (Lachman test and pivot-shift test). The details were shown in Table 1.

**Table 1:** Knee laxity assessment of included studies

Included studies	Arthrometer testing			Physical examination		Time from surgery to test/month
	Equipment	Flexion angle/°	Load level/N	Lachman test	Pivot test	
Engstrom 1993	Knee Laxity Tester; Stryker	20	NR	×	√	12–50
Ghalayini 2010	Stryker laxometer; Stryker	NR	NR	√	×	60
Grøntvedt 1995	KT-1000 arthrometer; MEDmetric	20	89	√	√	24
Liu 2010	KT-1000 arthrometer; MEDmetric	30	134	×	×	48–52
Nau 2002	Instrumented Laxity Tester; Telos	20	250	×	×	24
Pan 2013	KT-1000 arthrometer; MEDmetric	30	134	×	×	48–54
Pritchett 2009	KT-1000 arthrometer; MEDmetric	30	134	×	×	84–228

*NR* not reported

Any researches that failed to meet the inclusion criteria were excluded. In addition, a study was excluded if data from the same patients were reported in another study that had longer follow-up.

### Data extraction and quality assessment

Two reviewers independently performed data extraction and quality assessment. In case of discrepancies, any controversy was resolved by further discussion with the corresponding author. The extraction included the following: (1) the characteristics of included researches (author, publication date, study design, participants’ demography, sample size, and duration of follow-up); (2) the details of methodology (implant type and drilling technique); (3) the details of outcomes. In our research, Newcastle-Ottawa Scale (NOS) was used to assess quality for cohort study while Jadad scale was used to assess quality for randomized controlled trial (RCT) [19, 20].

### Statistical analysis

The meta-analysis was conducted using RevMan Manager 5.3 (Copenhagen: The Nordic Cochrane Centre, The Cochrane Collaboration, 2014). Using the same format, two reviewers independently collected data and crosschecked the results. Disagreements were discussed with the corresponding author and reached consensus in order to ensure accuracy.

Odds ratio (OR) with 95% confidence interval (CI) was calculated for dichotomous while mean difference (MD) with corresponding 95% CI was calculated for continuous outcomes. Statistical heterogeneity was assessed by calculating the heterogeneity index I^2^. When heterogeneity was significant (I^2^ > 50%), a Mantel-Haenszel analysis utilizing a random-effects model was used; otherwise a fixed-effects model was used when heterogeneity was considered as low (I^2^ ≤ 0.50). Funnel plots were used to test publication bias and a relatively symmetric funnel plot indicated inexistence of obvious publication bias. Sensitivity analysis was performed in order to maintain the stability of results.

## Results

### Article selection results

**Fig. 1:**
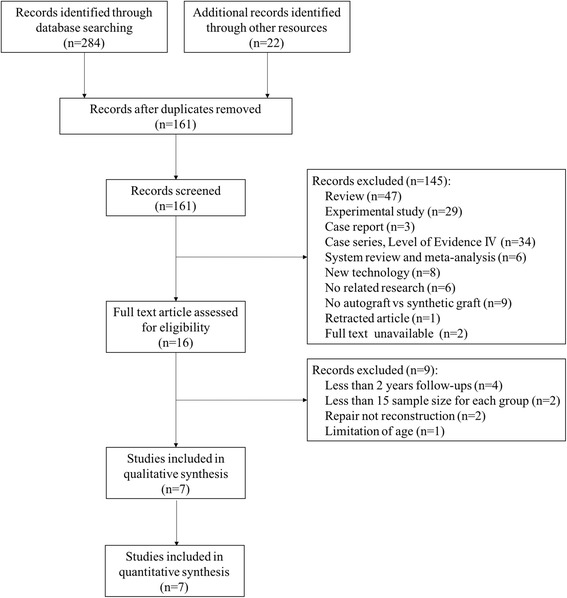
Flowchart of article selection process

Three hundred and six relevant articles were initially selected according to the search strategy (Fig. 1). There were 161 articles left after checking for duplicates by using the literature management software Endnote X7. One hundred and forty-five articles were removed by screening the title and abstract. After reviewing the full text, 9 articles were excluded through assessment for eligibility. Eventually, 7 articles were included in qualitative and quantitative synthesis [21–27].

### Characteristics of selected articles

All eligible studies were written in English from 1993 to 2013 (Table 2). Two studies were conducted in a North American country, and three studies were conducted in a European country. The other two studies were conducted in China. Among these studies, the synthetic graft used to compare with autograft included the Ligament Advanced Reinforcement System (LARS) artificial ligament (3 studies), the Leeds-Keio (LK) artificial ligament (2 studies), the Ligament Augmentation Device (LAD) (1 study) and the polyglycolic acid Dacron (PGA-Dacron) graft (1 study). The autograft used for comparison was BPTB (6 studies) and hamstring tendon (1 study). The rate of follow-up was ≥90% and the follow-up periods were ≥24 months in all included studies. The total sample size was 403 patients (autograft group: 206 patients; synthetic graft group: 197 patients). The release source and release date of each artificial ligament were shown in Table 3.

**Table 2:** Characteristics of Included Comparative Clinical Studies

Study	Journal	Implant		Followups (months)	Autograft			Synthetic graft			Outcome
		Autograft	Synthetic graft		No. of patients	Age	Gender (Male/Female)	No. of patients	Age	Gender (Male/Female)	
Pan 2013	Eur J Orthop Surg Traumatol	BPTB	LARS	50 (48–54)	30	33.9	19/11	32	35.9	25/7	Anterior laxity; IKDC score; Lysholm score; Tegner score
Ghalayini 2010	Knee	BPTB	LK	60	26	30.9	19/7	24	31.7	21/3	Anterior laxity; IKDC score; Lysholm score; Tegner score; one-hop test
Liu 2010	Int Orthop	HT	LARS	49 (48–52)	32	32	24/8	28	36	21/7	Anterior laxity; IKDC score; Lysholm score; Tegner score
Pritchett 2009	J Knee Surg	BPTB	PGADacron	138 (84–228)	35	25	24/11	35	26	23/12	Anterior laxity; IKDC score; Lysholm score; KOOS
Nau 2002	J Bone Joint Surg Br	BPTB	LARS	24	27	30.9	15/12	26	31.0	21/5	Anterior laxity; IKDC score; Tegner score; KOOS
^a^Grøntvedt 1995	Scand J Med Sci Sports	BPTB	LAD	24	26	NR	NR	22	NR	NR	Anterior laxity; Lysholm score; Tegner score; Isokinetic strength
Endstrom 1993	Clin Orthop Relat Res	BPTB	LK	28	30	23.8	14/16	30	23.4	21/9	Anterior laxity; IKDC score; Lysholm score; Tegner score; Muscle performance

*BPTB* bone-patellar tendon-bone, *LARS* ligament advanced reinforcement system, *LK* Leeds-Keio synthetic graft, *HT* hamstring tendon, *LAD* ligament augmentation device, *PGA-Dacron* polyglycolic acid-Dacron, *IKDC* International Knee Documen- tation Committee, *KOOS* Knee Injury and Osteoarthritis Outcome Score, *NR* not reported

^a^The mean age and the gender distribution were not described separately in this study. The mean age of the patients was 25 years (range 15–42). There were 18 men and 30 women altogether including in this research

**Table 3:** Details of each artificial ligament in included study

Included studies	Synthetic product name	Release source	Release date
Engstrom 1993	Leeds-Keio graft	Neoligaments, Leeds, UK	1980
Ghalayini 2010	Leeds-Keio graft	Xiros plc formerly Neoligaments Ltd., Leeds, UK	1980
Grøntvedt 1995	LAD	3 M Company, St. Paul, Minnesota, USA	1980
Liu 2010	LARS artificial ligament	Surgical Implants and Devices, Arc-sur-Tille, France	1985
Nau 2002	LARS artificial ligament	Surgical Implants and Devices, Arc-sur-Tille, France	1985
Pan 2013	LARS artificial ligament	Surgical Implants and Devices, Arc-sur-Tille, France	1985
Pritchett 2009	PGA-Dacron graft	Surgitex, Southfield, Mich	NR

*LAD* ligament augmentation device, *LARS* ligament advanced reinforcement system, *PGA-Dacron* polyglycolic acid-Dacron, *NR* not reported

The synthetic grafts were divided into two groups (Group 1: early generation; Group 2: new generation) for analysis. In this study, the early generation of the artificial ligaments contained the LK artificial ligament and the LAD, while the new generation included the LARS artificial ligament and the PGA-Dacron graft [2, 26]. Among all included articles, 4 articles were related to the new generation and 3 articles were related to the old generation (Table 2).

### Quality of selected articles

Assessment of the methodological quality revealed that there were four RCTs (Level I). Two studies were retrospective comparative studies (Level III) and one study was non-randomized prospective comparative study (Level II). Among these four RCTs, only one article was of high quality with scores ≥4 while the other three articles were of low quality with scores ≤3 according to Jadad scale (Table 4). Assessed by NOS scale, two retrospective studies and one prospective study were of high quality. All demographic data were compared between two groups and showed no significant difference in eligible studies.

**Table 4:** Quality assessment of included studies

Study	Level of evidence	Type	NOS	Jadad scale
Pan 2013	III	Retrospective study	7	
Ghalayini 2010	I	RCT		5
Liu 2010	III	Retrospective study	7	
Pritchett 2009	II	Prospective study	7	
Nau 2002	I	RCT		3
Grøntvedt 1995	I	RCT		1
Endstrom 1993	I	RCT		1

*NOS* Newcastle-Ottawa Scale, *RCT* randomized controlled trial

### Meta-analysis

#### Instrumented laxity

**Fig. 2:**
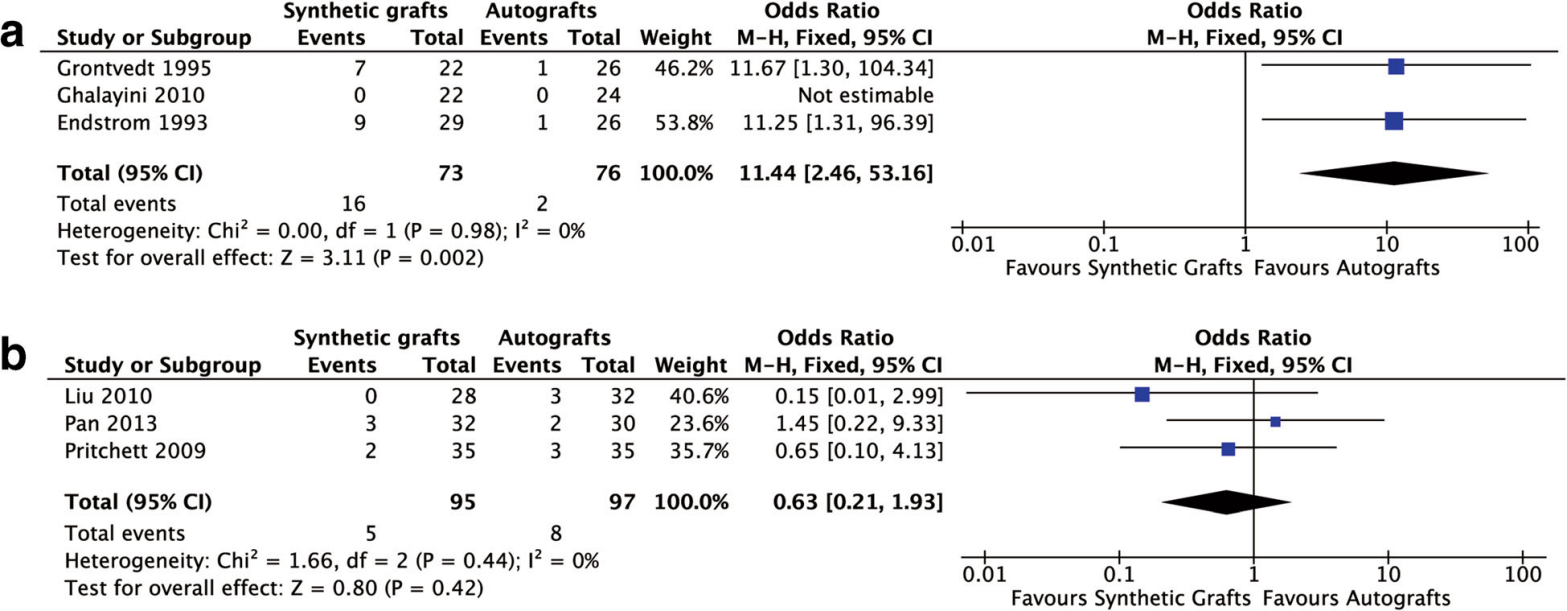
Forest plot of the instrumented laxity. **a** the early generation of synthetic grafts; **b** the new generation of synthetic grafts

All included studies tested instrumented laxity. The study of Nau et al. was excluded for providing quantitative data other than grade data of instrumented laxity (> 5 mm or ≤5 mm), which could not be compared with other studies [22]. No heterogeneity was found among the studies. Using the fixed-effects model in analysis, the early generation of synthetic grafts had a significant difference in knee laxity compared with autografts and the synthetic graft had a poorer result (OR = 11.44; 95% CI: 2.46, 53.16; *p* = 0.98; I^2^ = 0%; Fig. 2a). Conversely, the new generation of synthetic graft showed no significant difference in knee laxity compared with autografts (OR = 0.63; 95% CI: 0.21, 1.93; *p* = 0.44; I^2^ = 0%; Fig. 2b).

#### Physical examinations

Two studies assessed the anterior stability by Lachman test and two studies evaluated the rotational stability through pivot-shift test (Table 1). All included studies were related to the early artificial ligaments (LK artificial ligament and LAD). The Lachman test showed a poorer result in the early synthetic grafts than in the autografts (OR = 0.02, 95% CI: 0.00, 0.41), indicating a worse anterior stability. The result of pivot-shift test was poor in early synthetic grafts (OR = 0.03, 95% CI: 0.01, 0.16), documenting a worse rotational stability comparing to autografts.

#### International knee documentation committee (IKDC) grades

**Fig. 3:**
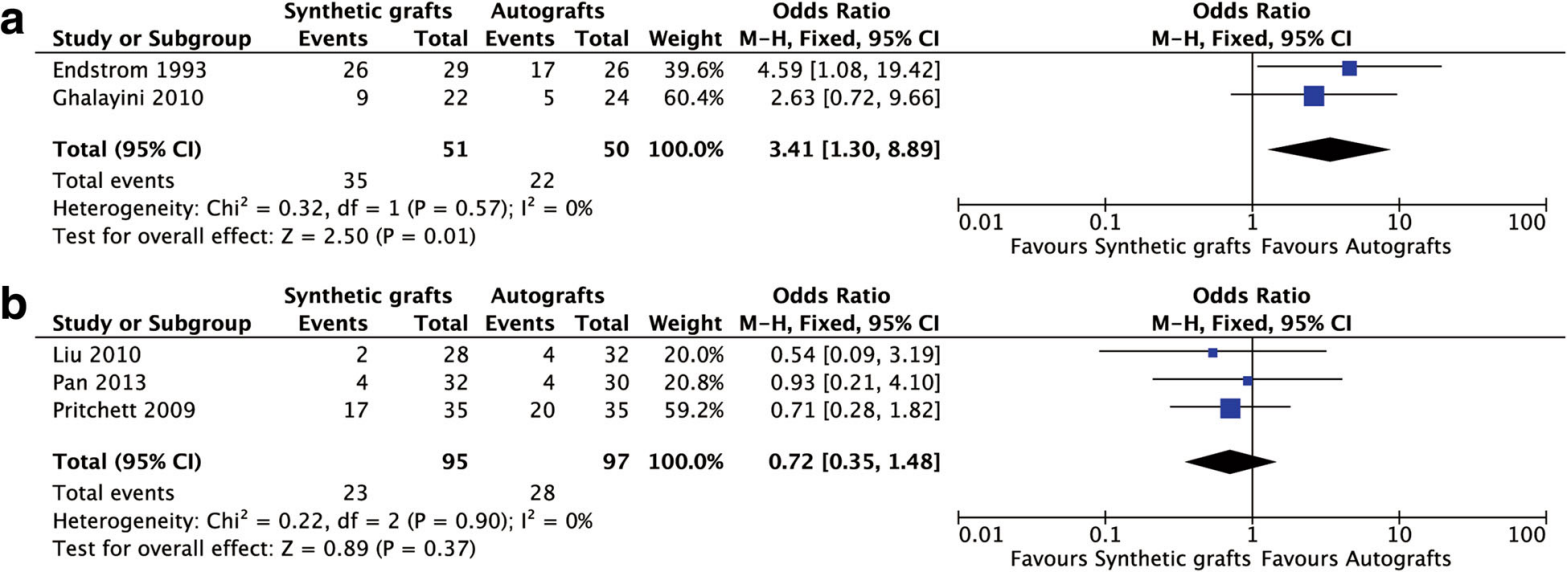
Forest plot of the IKDC grades. **a** the early generation of synthetic grafts; **b** the new generation of synthetic grafts

Six studies reported postoperative IKDC grades but the study of Nau et al. was excluded for providing the different type of categorical data comparing to other included studies [22]. No heterogeneity was found and a fixed-effects model was used to analysis (Fig. 3). There were 51 patients in the early synthetic graft group and 50 patients in the autograft group. The early synthetic grafts (LK, LAD) had worsen IKDC grades (OR = 3.41; 95% CI: 1.30, 8.89; *p* = 0.57; I^2^ = 0%; Fig. 3a). Altogether 95 cases in the new synthetic graft group and 97 cases in the autograft group were reported. The new synthetic grafts (LARS) had no difference in IKDC grades compared to autografts (OR = 0.72; 95% CI: 0.35, 1.48; *p* = 0.90; I^2^ = 0%).

#### Lysholm scores

Six eligible studies tested postoperative Lysholm scores but the results of two studies could not be analyzed in meta-analysis. One was excluded due to lack of standard deviation and the other was due to suppling Lysholm scores as grade data other than quantitative data [21, 24]. Three studies were in Group 2 while only one study was in Group 1. There were altogether 95 cases in Group 2 and 97 cases in the autograft group. Heterogeneity was not found among these three studies and a fixed-effects model was used (*p* = 0.88; I^2^ = 0%), showing no significant difference in the Lysholm scores between two groups (OR = 1.80; 95% CI: -0.52, 4.13).

#### Tegner scores

Six studies reported Tegner scores but only 3 studies applied mean scores and standard deviations [23, 25, 27]. The rest three studies documented there was no significant difference between two groups in their longest follow-up time. Two studies were related to the new generation of the synthetic grafts and one study were focused on the old generation. Heterogeneity was not significant and a fixed-effects model was used, no significant difference occurred in new synthetic grafts and autografts (OR = 0.40; 95% CI: -0.09, 0.89).

#### Complications

**Fig. 4:**
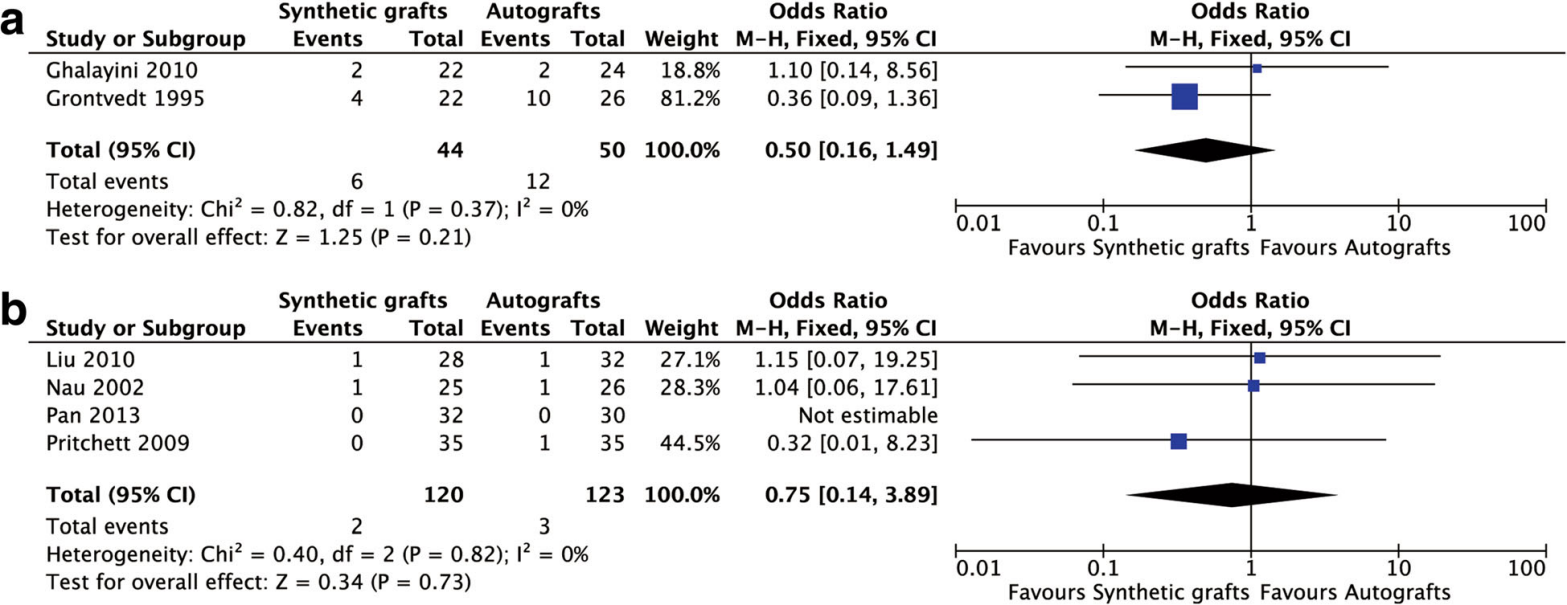
Forest plot of the complications of anterior cruciate ligament reconstruction. **a** the early generation of synthetic grafts; **b** the new generation of synthetic grafts

Six studies evaluated complications of ACL reconstruction. The study conducted by Endstrom et al. did not report the complications after ACL reconstruction and was excluded for analysis. No heterogeneity was found and a fixed-effects model was used (I^2^ = 0%; Fig. 4). Altogether 44 patients were included in the early synthetic graft group and 50 patients were included in the compared group. No significant difference was found in the rate of complications between two groups (OR = 0.50; 95% CI: 0.16, 1.49; Fig. 4a). Similarly, no significant difference occurred in the new synthetic grafts and autografts (OR = 0.75; 95% CI: 0.14, 3.89; Fig. 4b).

Sensitivity analysis indicated that the study with regard to four-strand HT graft had no obvious deviation compared to other studies concerning about BPTB in evaluation of knee laxity, patient-oriented outcomes and the rate of complications.

### Publication bias

**Fig. 5:**
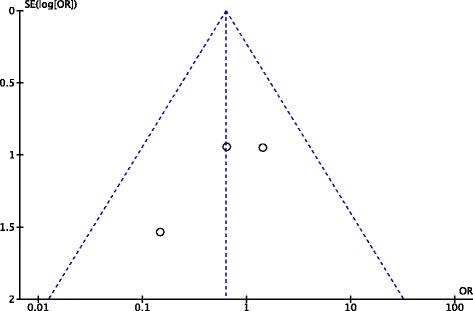
Funnel plot of publication bias for instrumented laxity in the new generation synthetic graft group

**Fig. 6:**
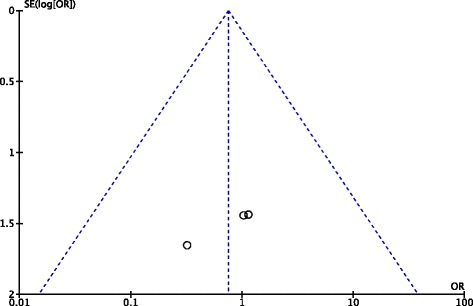
Funnel plot of publication bias for complications in the new generation synthetic graft group

Funnel plots of instrumented laxity and complications were used to evaluate the publication bias, showing the lack of obvious bias among the eligible studies related to new synthetic grafts according to a relative symmetric funnel plot (Figs. 5 and 6).

## Discussion

The key findings of present meta-analysis indicated that, in general, the patient-oriented outcomes and the rate of complications of ACL reconstruction with synthetic grafts were not significantly different from those with autograft, especially for new generation synthetic grafts (LARS and PGA- Dacron). However, with regard to knee laxity, ACL reconstruction with early artificial grafts had obviously poorer knee laxity from those with autografts (95% CI: 1.03, 4.72) while new artificial grafts showed no significant difference with autografts (95% CI: 0.21, 1.93).

The LK artificial ligament was a polyester mesh-like structure intended as a scaffold for soft tissue ingrowth [28]. The LAD, a band-like braid of polypropylene, was designed to protect the autogenous graft from excessive stresses [29]. Murray et al. reported that 28% of the group were known to have ruptured the LK ligament and 56% had increased laxity compared to the opposite normal knee at a 10–16 year follow-up [30]. A study conducted since 1983, included 856 patients accepted ACL reconstruction with LAD, showed 63 cases of complications and 73 cases of re-surgery [31]. Long-term follow-up results documented both the LK artificial ligament and the LAD were not suitable as an ACL substitute [30–32]. Moreover, the LAD caused effusions and reactive synovitis in the knee for provoking inflammatory reactions, and was found to delay maturation of autogenous graft [33]. The knee laxity and the IKDC grades were significantly different from autografts and early artificial ligaments, indicating that the short-term outcomes of early artificial ligaments were worsen than autografts. The results of our research for early artificial ligaments were consistent with previous studies. It was not suggested to use early synthetic grafts including the LK artificial ligament and the LAD due to their poor follow-up outcomes.

The LARS artificial ligament was made of polyethylene terephthalate, divided in two parts (intra-articular part and extra-articular part) [34]. Intra-articular part was composed of longitudinal external rotation fibers without transverse fibers as an imitation of ACL anatomic structure while extra-articular part was weaved by longitudinal and transverse fibers in order to avoid ligament deformation. Dericks et al. reported encouraging results in 220 cases of ACL reconstruction used LARS artificial ligament with a mean follow-up of 2.5 years [35]. In 2013, Parchi reported no case of complications and only one case of mechanical graft rupture after using LARS artificial ligament for ACL reconstruction at a mean follow-up of eight years [36]. In 2015, a study with a minimum follow-up of 10 years, showed almost half of the patients (8/18) were subjectively not satisfied with the surgical result using LARS artificial ligament [7]. The clinical outcomes were appealing at short-term but controversy at long-term [36–38]. In our research, 3 studies compared LARS artificial ligament with autografts, showing no significant difference in knee laxity, functions and the rate of complications [22, 25, 27]. The outcomes of LARS artificial ligament used in ACL reconstruction were appealing at least in short-term follow-up. Another new synthetic graft called PGA-Dacron graft, consisted of synthetic braided ligament made of 75% degradable PGA filaments and 25% non-degradable Dacron thread, showed a satisfied result compared to autograft including knee laxity, range of motion, patient-oriented questionnaires, muscle performance, degenerative changes of knee, and the rate of failure and complications [26].

Complications occurred in the autograft group were infection, patellofemoral pain, recurrent effusion and extension loss. In the synthetic graft group, complications included interference screw-related problems (pain and screw loosening), patellofemoral pain and extension loss. There were altogether 12 cases in the autograft group and 8 cases in the synthetic graft group. Extension loss was the most common complication in included studies and it might be associated with graft impingement and a formation of cyclops [39, 40]. Graft impingement was mainly caused by malposition of femoral bone tunnel and a “cyclops” was a fibrous nodule caused by proliferation of fibrovascular tissues similar to a healing scar after ACL reconstruction [41, 42]. The synthetic grafts were located in a non-anatomic but isometric placement while the autografts were usually located in an anatomic placement. The results of complications showed no significant difference between these two location methods.

Some studies documented that subjective outcomes were not correlated with objective outcomes including instrumented laxity test and clinical examination [43]. Among these included studies, three of them showed difference in objective parameters but no significant difference in patient-oriented outcomes [21, 22, 24]. Meanwhile, the opposite circumstance did not appear (similar in objective outcomes but different in subjective outcomes). Kraeutler et al. suggested that patient satisfaction is the most important measurable index for the outcomes of ACL reconstruction [13]. Only the overall IKDC grades showed better results in the autografts than in the early synthetic grafts and the rest indicators for patient satisfaction showed no significant difference between groups. However, it was still well recognized that a KT-1000 side-to-side difference of >5 mm was defined as a clinical failure [37]. Both objective parameters and subjective outcomes shoulder be considered for assessment of ACL reconstruction.

The limitations of this study were as follows: (1) Until now, there was still lack of high-quality RCT or large-scale multi-center retrospective comparable studies to prove the effectiveness of artificial ligaments compared to autografts. (2) The follow-up time was not sufficiently long for evaluation of ACL reconstruction. (3) In the included studies, the types of grafts used in ACL reconstruction were not the same (Hamstring tendon, BPTB, LK, LAD, LARS and PGA-Dacron). (4) The data included in the research did not cover all included studies due to the lack of relative data.

## Conclusions

The outcomes of new generation of artificial ligaments are similar to autografts in terms of knee laxity, patient-oriented outcomes and the rate of complications at a short- to mid-term follow-up. However, the early artificial ligaments (LK, LAD) are not suggested for ACL reconstruction according to worse outcomes in knee laxity and functions compared to autografts.

## Abbreviations

ACL: Anterior cruciate ligament
BPTB: Bone-patella tendon-bone
CI: Confidence interval
IKDC: International Knee Documentation Committee
ISRCTN: International Standard Randomised Controlled Trial Number
LAD: Ligament Augmentation Device
LARS: Ligament Advanced Reinforcement System
LK: Leeds-Keio
MD: mean difference
NOS: Newcastle-Ottawa scale
OR: Odds ratio
PGA-Dacron: polyglycolic acid Dacron
PRISMA: Preferred Reporting Items for Systematic Reviews and Meta-Analyses
RCT: Randomized controlled trial
